# Supplementary material for: E-Learning versus Face-to-Face Methodology for Learning Antimicrobial Resistance and Prescription Practice in a Tertiary Hospital of a Middle-Income Country
Source: Antibiotics (Basel). 2022 Dec 16;11(12):1829. doi: 10.3390/antibiotics11121829 (PMC9774894; doi:10.3390/antibiotics11121829)
Supplement: Supplementary file 1 [file antibiotics-11-01829-s001.zip › antibiotics-2050561-supplementary.pdf]

Knowledge, attitude and referred practice assessment test on Antimicrobial Resistance and Good Prescription Practices.

LAST 4 DIGITS OF IDENTITY CERTIFICATE: ____________________

QUESTIONNAIRE OF KNOWLEDGE, ATTITUDES AND REFERRED PRACTICES REGARDING THE USE OF ANTIBIOTICS

Title: COURSE ON ANTIMICROBIAL RESISTANCE AND GOOD PRESCRIPTION PRACTICES

Instruction:

This questionnaire is applied within the framework of the study called: Design and evaluation of an educational methodological strategy to improve knowledge, attitudes and practices on antimicrobial resistance and good prescription practices in doctors of the Intensive Care and Internal Medicine services of the Hospital. of Specialties Eugenio Espejo de Quito, from October 2020 to March 2022., carried out by Dr. Paulina Armas.

The objective of this questionnaire is to assess the knowledge, attitudes and practices referred to, so be completely honest in your answers, remember that it is absolutely confidential.

The questionnaire must be completed by dd/mm/yyyy at 23:59.

Questions about the participant's perception of the medical practice and their patient's perception.

Sociodemographic characteristics of the participants.

How old are you?
Gender: Male Female
In which hospital department do you work?<br>a) Intensive therapy<br>b) Internal Medicine
What occupation do you have?<br>a) Resident doctor<br>b) Postgraduate doctor

c) Medical specialist
How long have you worked in your current profession? Specify in months and years

Characteristics of the use of antimicrobials

For you, what is the usual source of information about antibiotics or other drugs:<br>a) Literature provided by medical representatives<br>b) Information obtained from other doctors<br>c) Internet or other online platform
What proportion of patients who come to your doctor's office seek antibiotics?<br>a) none of my patients<br>b) some of my patients<br>c) most of my patients<br>d) all my patients
If a patient feels they need antibiotics, they will arrange to get them from the pharmacy without a prescription, even when they were not prescribed.<br>a) Yes<br>b) Nope
In your practice, how often would you say your empirical coverage correlates with microbiology laboratory susceptibility reports?<br>a) Always<br>b) Very often<br>c) Frequently<br>d) Rarely<br>e) Very rarely<br>f) Never

Conditions of the participants when prescribing an antimicrobial

	Never	Sometimes	Most of the time	Always
How often do you prescribe antibiotics in your practice?				
How often do you prescribe or provide an antibiotic for a cold or sore throat?				

How often do you review your decision to prescribe antibiotics with a colleague?				
How often do you explain the proper use of antibiotics to your patients?				

Module 1: Antimicrobial Resistance

Perception questions

1	Of the following factors, check the one or those that you think contribute to the development of antibiotic resistance?	Yes	No
	Widespread use of antibiotics.		
	Inappropriate empiric choice of antibiotics.		
	Inadequate duration of antibiotic therapy.		
	Prescribe broad-spectrum antibiotics when equally effective narrow-spectrum antibiotics are available.		
	Inadequate handwashing		
	Inappropriate restrictions on the prescription of antibiotics.		
	Poor access to information on local patterns of antibiotic resistance.		
	Lack of guidelines on the use of antibiotics.		
	Random mutations in microbes.		
	Patient demands and expectations regarding antibiotics.		
	Use of antibiotics in the livestock industry.		
	Role of pharmaceutical companies in advertising and promoting the use of antibiotics.		

Knowledge questions

		Yes	No
1	Is antibiotic resistance a phenomenon by which a bacterium loses its sensitivity to an antibiotic?		
two	Should general knowledge about antibiotic resistance be taken into account when prescribing antibiotics to an individual patient?		
3	Does the prescription of an antibiotic to a patient influence the possible appearance of resistance?		
4	Is the use of antibiotics in animals an important cause of the emergence of new resistance to pathogens in humans?		
5	Two of the main causes of the emergence of antibiotic resistance are patient self-medication and improper use of antibiotics?		
6	Is Methicillin-Resistant Staphylococcus aureus susceptible to?<br>a) Amoxicillin/clavulanic acid<br>b) Cefotaxime<br>c) Ceftriaxone<br>d) None of these antibiotics		

Attitude questions

		In agreement	In disagreement
1	A training program on antibiotic resistance is needed.		
2	I believe that I can do something to combat antibiotic resistance in my practice.		
3	Antibiotic resistance is a problem all over the world		
4	Antibiotic resistance is a problem for my patients in my daily practice		

Practice questions

1	In a patient who is responding clinically to current antibiotic therapy, what is your response to a culture report indicating that the isolated organisms are resistant to that antibiotic regimen?

	a) I switch to appropriate antibiotics according to the report<br>b) I am still on current antibiotics.<br>c) I add one of the susceptible antibiotics to the current regimen
2	If the culture report shows an isolate that is sensitive to my current antibiotics, but also to a narrower spectrum antibiotic<br>a) I continue current therapy<br>b) I switch to narrow spectrum antibiotic
3	A 62-year-old male patient who is hospitalized for atypical pneumonia with suspected COVID 19. Per protocol, a urine culture is requested by catheter puncture (7 days before the catheter is placed). The microbiological report reports the isolation of >10 5 CFU of *Klebsiella pneumoniae* , producer of KPC-type carbapenemases. You make the following decision:<br>a) Start treatment with maximum dose meropenem.<br>b) He considers that there is no clinic of urinary tract infection, requests to change the probe and send a new sample.<br>c) Start treatment with Fosfomycin<br>d) Start treatment with Colistin plus Tigecycline
4	A 45-year-old male patient underwent surgery for appendicitis, on the second day he presented fever spikes, tachycardia and purulent secretion was evident in the surgical wound, so he sent this sample for culture and received the following report: Escherichia coli producing beta-lactamase of the extended ESBL. You make the decision to start treatment with:<br>a) Vancomycin plus amikacin.<br>b) Vancomycin plus Meropenem.<br>c) Meropenem plus colistin.<br>d) Meropenem plus amikacin
5	Do you use the information provided by the microbiological report to guide your therapeutic choice of the best antimicrobial that you can prescribe according to the case?<br>a) Yes<br>b) Nope

Module 2: ***Good antimicrobial prescribing practices***

Perception questions

1	In the treatment of infection, how would you rate the usefulness of each of these sources of knowledge?	Useful	Not useful

	Clinical practice guidelines.		
	Previous clinical experience.		
	Continuing education courses.		
	Others, for example, contribution from specialists (microbiologists, infectious disease specialists, etc.).		
	Peer contribution (of the same specialization).		
	Data collected through the Internet		

Knowledge questions

		Yes	No
1	Are antibiotics useful for bacterial infections (Example: typhoid)?		
2	Are antibiotics useful for viral infections (Example: flu)?		
3	Are antibiotics indicated to reduce the symptoms of pain and inflammation?		
4	In a primary care setting, should one wait for microbiology results before treating an infectious disease?		
5	Which of the following antibiotics is contraindicated in pregnancy?<br>a) Amoxicillin<br>b) Ciprofloxacin<br>c) Gentamicin		
6	Which of the following antibiotics has the best activity against anaerobes?<br>a) Ciprofloxacin<br>b) Trimethoprim/Sulfamethoxazole<br>c) Metronidazole		

Attitude questions

		In agreement	In disagreement

1	Do you think you have enough sources of antibiotic information when you need it?		
2	Do you think it is difficult to select the correct antibiotic for a particular disease?		
3	Do you think that prescribing antibiotics is harmful to patients if they are not needed?		
4	Do you think knowledge of antibiotics is important to you in your role as a healthcare provider?		
5	Would you like to access educational programs on antibiotic prescription?		
6	Do you think antibiotics are used excessively in Ecuador?		
7	Do you think patient demands for antibiotics contribute to overuse?		
8	Do you think the use of antibiotics without a prescription should be more closely monitored?		
9	If in doubt, do you think it is preferable to use a broad-spectrum antibiotic to ensure that the patient is cured of an infection?		

Practice questions

1	A 40-year-old woman comes to you complaining of 4 days of loose, watery stools with 1-2 episodes of vomiting. No history of fever. What antibiotic will you recommend?<br>a) Ciprofloxacin<br>b) Metronidazole<br>c) Trimethoprim-sulfamethoxazole<br>d) The use of antibiotics is not necessary. Oral rehydration only.
2	I frequently prescribe an antibiotic in situations where it is impossible for me to follow the patient systematically.<br>a) Real<br>b) Fake
3	In situations of doubt as to whether an illness could be of bacterial etiology, it is preferable to prescribe an antibiotic.<br>a) Real<br>b) Fake
4	Sometimes I prescribe antibiotics so that patients continue to trust me.

	a) Real<br>b) Fake
5	A patient has been prescribed a full course of antibiotics. After 48 hours the patient is asymptomatic, is it correct then to suspend the antibiotic within another 48 hours?<br>a) Real<br>b) Fake
6	A 4-year-old boy had diarrhea for 4 days (3 stools per day). He had no fever during the last days or in consultation. What is your choice of treatment?<br>a) oral amoxicillin<br>b) Trimethoprim / Sulfamethoxazole<br>c) Amoxicillin/clavulanic acid<br>d) No antibiotics, only oral rehydration
7	A 6-year-old boy has a fever (38°C), a runny nose, and a sore throat for two days. On visual inspection, the throat is reddish. What is your choice of treatment?<br>a) Trimethoprim / Sulfamethoxazole<br>b) oral amoxicillin<br>c) Amoxicillin/clavulanic acid<br>d) no antibiotics

Course Perception Questions

a) What did you like about the course?
b) What did you not like about the course?
c) What would you recommend for a future course?
d) Course rating:

Please rate how successful you think this course was, based on the following description:<br>1-3 below expectations,<br>4-6 within expectations,<br>7-9 above expectations
Please assign a score for your self-assessment (in knowledge, attitudes and practices) within this course, according to the following description:<br>1-3 below expectations;<br>4-6 within expectations;<br>7-9 above expectations

Gratitude:

Sincere thanks are extended to whoever filled out this questionnaire, your honest responses are very important to the investigation.
